# Cold acclimation alleviates photosynthetic inhibition and oxidative damage induced by cold stress in citrus seedlings

**DOI:** 10.1080/15592324.2023.2285169

**Published:** 2023-11-28

**Authors:** Chao Xu, Yuting Wang, Huidong Yang, Yuqing Tang, Buchun Liu, Xinlong Hu, Zhongdong Hu

**Affiliations:** a Nanchang Key Laboratory of Germplasm Innovation and Utilization of Fruit and Tea, Jiangxi Academy of Agricultural Sciences, Nanchang, P. R. China; b Institute of Environment and Sustainable Development in Agriculture, CAAS, Beijing, P. R. China

**Keywords:** Citrus, cold acclimation, cold stress, oxidative stress, photosynthesis

## Abstract

Cold stress seriously inhibits plant growth and development, geographical distribution, and yield stability of plants. Cold acclimation (CA) is an important strategy for modulating cold stress, but the mechanism by which CA induces plant resistance to cold stress is still not clear. The purpose of this study was to investigate the effect of CA treatment on the cold resistance of citrus seedlings under cold stress treatment, and to use seedlings without CA treatment as the control (NA). The results revealed that CA treatment increased the content of photosynthetic pigments under cold stress, whereas cold stress greatly reduced the value of gas exchange parameters. CA treatment also promoted the activity of Rubisco and FBPase, as well as led to an upregulation of the transcription levels of photosynthetic related genes (*rbcL* and *rbcS*)，compared to the NA group without cold stress. In addition, cold stress profoundly reduced photochemical chemistry of photosystem II (PSII), especially the maximum quantum efficiency (F_v_/F_m_) in PSII. Conversely, CA treatment improved the chlorophyll *a* fluorescence parameters, thereby improving electron transfer efficiency. Moreover, under cold stress, CA treatment alleviated oxidative stress damage to cell membranes by inhibiting the concentration of H_2_O_2_ and MDA, enhancing the activities of superoxide dismutase (SOD), catalase (CAT), ascorbic acid peroxidase (APX) and glutathione reductase (GR), accompanied by an increase in the expression level of antioxidant enzyme genes (*CuZnSOD1*, *CAT1, APX* and *GR*). Additionally, CA also increased the contents of abscisic acid (ABA) and salicylic acid (SA) in plants under cold stress. Overall, we concluded that CA treatment suppressed the negative effects of cold stress by enhancing photosynthetic performance, antioxidant enzymes functions and plant hormones contents.

## Introduction

Plants have evolved many positive and effective multifaceted acclimation strategies to cope with their changing surroundings during their growth and development, especially the unfavorable environmental stresses.^1,2^ Cold stress is one of the important environmental stress factors, which not only affects the nutritional and reproductive growth of plants, but also limits their yield. Cold acclimation refers to the process in which plants acquire stronger cold resistance after being treated with non-lethal low temperatures for a short period of time. Exposure of plants to non-lethal low temperatures for a short period of time modulates their physiological and biochemical processes, thereby inducing cold stress resistance, which is called cold acclimation (CA).^3^ To alleviate the adverse effects of cold stress, plants have evolved CA mechanisms, which is associated with physiological and biochemical alterations, including modifications of photosynthesis or photosynthetic electron transport, reactive oxygen species variations, osmotic regulation, as well as changes of gene expression and metabolism.^4,5^

Photosynthesis is a crucial physiological process, through which plants convert carbon dioxide and water into complex organic compounds and release oxygen.^6^ The photosynthetic apparatus in plants incorporates diverse components, such as photosynthetic pigments for light absorption, photosystems and the light reactions for NADPH and ATP generation, and the dark reactions for CO_2_ assimilation.^7^ Among these, PSII is considered as the most cold-sensitive component in the photosynthetic apparatus. Cold stress reduces the photosynthetic capacity by inhibiting PSII activity, which leads to a decrease in its F_v_/F_m_ and an increase in non-photochemical quenching (NPQ).^8^

When plants exposed to cold stress, the concentration of chlorophyll in its leaves also decreases. In addition, the chlorophyll biosynthesis is affected, resulting in an imbalance between the PSII and antenna complex.^9^ As an urge to increase photon capture, plants under cold stress typically accumulate higher concentrations of auxiliary pigments, such as chlorophyll b and carotenoids, compared to chlorophyll a.^7^ Photosynthetic enzymes including Rubisco, directly participate in CO_2_ fixation, which are vulnerable to low temperatures.^10^ The synthesis rate of ribose-1,5-diphosphate (RuBP) and the carboxylation sites of Rubisco are highly sensitive to temperature changes.^11^ There is considerable evidence to suggest that genes like *rbcL* and *rbcS* drive the basic function of CO_2_ assimilation. CA can limit low-temperature induced photoinhibition and promote PSII recovery.^12^ For instance, CA reduces susceptibility to photoinhibition by causing several metabolic changes and producing variations in chloroplast levels that may restore energy balance. Moreover, CA can also enhance the capacity of plants to maintain metabolism under low temperature by making the main quinone electron acceptor QA more oxidized.^13,14^ More importantly, CA treatment enhances a series of Calvin cycle enzymes (such as FBPase and Rubisco) activities and greatly improves D1 synthesis ability in cucumber seedlings under low temperature conditions.^15^

Chlorophyll a fluorescence analysis is a nondestructive, efficient and sensitive tool for monitoring the mechanisms of photosynthetic apparatus in diverse environments.^16^ and also use to assess the consequences of plant stress induced photosynthesis, which allows to get detail information about the photosynthetic process without damaging the experimental sample.^17,18^ The analysis of OJIP kinetics, referred to as the “JIP-test”, is a useful technique for evaluating the function of the photosystem, which may also be helpful for providing direct information on the status and function of PSII reaction centers, light-harvesting antenna complexes, and the donor and receptor sides of PSII.^19^ Further, JIP-test has been used to study stress alleviation. In current study, the JIP-test has been used to obtain detailed information on the negative effects of cold stress on different sites in photosynthetic machinery and to reveal that whether the stress effects alleviated in the CA treatment plants of citrus, and the photosynthetic performance based on light absorption in CA group was higher than that of the cold stress group.

The most severe sequel of cold stress is the excessive production of reactive oxygen species (ROS), which can lead to oxidative damage to plant cells at high concentrations. To minimize ROS concentration and control redox homeostasis, plants have developed an array of efficient defense strategies, including effective enzymatic antioxidants and non-enzymatic antioxidants, such as SOD, CAT, APX, glutathione GR, DHAR, AsA and GSH^20^. Beside, CA also induces other biochemical mechanisms, such as transcription of genes encoding stress proteins, non-structural carbohydrate content, and lipid composition.CA increases the proportion of non-structural carbohydrates (Starch and sugar) contents in tobacco leaves and improves the activity of antioxidant enzymes and accumulating antioxidants, alleviating the damage of membrane peroxidation under cold stress.^3^ Cold stress regulates Ca^2+^ channels, induce Ca^2+^ transients, cross-talks with nitric oxide (NO), ROS and mitogen-activated protein kinases (MAPKs) signaling pathways, thereby enhancing plant cold resistance.^21^ Meanwhile, CA is a polygenic inheritance that involves a large number of gene interactions, among which *CBF* transcription factors are the core of cold acclimation regulation.^22,23^ There are three tandemly arranged *CBF* genes in *Arabidopsis thaliana*, and overexpression of *CBF1*, *CBF2*, and *CBF3* can significantly improve the cold resistance in plants.^23^ Zhao et al^24^ revealed that the *CBF* genes regulate the expression of 414 downstream cold-responsive (COR) genes, of which 346 are CBF-activated genes and 68 are CBF-repressed genes, enhancing the cold resistance of transgenic plants.^25^ Moreover, the studies have found that CA also improves the cold tolerance of plants by regulating hormone metabolism like GA_3_, CTK and IAA or stimulating the transcription level of potential antifreeze protein genes.^26,27^

Citrus is a typical tropical and subtropical fruit tree, which is susceptible to the effects of cold and freezing^28^. During early spring, when citrus seedlings are planted in field, they are often damaged by low temperatures or frost, leading to slow growth and development, and even death. Therefore, before seedlings transplantation, a portion of the greenhouse plastic film is usually uncovered to maintain a stable temperature of around 7–11, used for CA treatment, thereby enhancing the cold tolerance of citrus seedlings. This study assessed the photosynthetic performance and antioxidant enzyme activity of citrus seedlings under and cold stress with/without CA treatment, thereby revealing the potential physiological and biochemical mechanisms underlying these phenomena. Besides, the mechanism of oxidative damage and energy transfer in PSII reaction center of citrus leaves under cold stress conditions was also analyzed. Our research results provide necessary theoretical support for improving the cold tolerance of citrus seedlings and developing reasonable cultivation measures.

## Materials and methods

### Experimental materials and treatments

This study used 3-year-old, healthy, and consistently growing Nanfeng Tangerine (*Citrus reticulata* Blanco cv. Kinokuni) seedlings as experimental materials, which is the widely cultivated citrus variety in Nanfeng County, Jiangxi Province, China. The seedlings were planted into the pot with a diameter of 50 cm both at the top and bottom, and a height of 70 cm, filled with citrus specialized cultivation substrate with an organic matter content of 3%-5%, an available phosphorus content of 0.09%, an available potassium content of 1%, a hydrolyzed nitrogen content of 0.2%, and a pH value between 5.5 and 6.5. Before the experiment, healthy and uniform citrus seedlings were cultivated in the artificial climate chamber for 7d, with the temperature is 28/18°C (day/night), and the light intensity is 800 μmol m^−2^ s^−1^, the photoperiod is 12/12 hours (day/night) and the relative humidity is about 65%.

After pre-treatment was completed, all citrus seedlings were then randomly arranged into two groups. One group of seedlings were grown in the artificial climate chamber (A1000, Conviron, Canada) and undergo cold acclimation treatment at a temperature of 7/10°C (night/day) for 3d was recorded as CA. The other group of seedlings that were not cold acclimation was recorded as NA. Both the CA and NA groups of seedlings were then subjected to cold stress treatment at a low temperature of 4/7°C (night/day) (recorded as CA + 4/7°C stress and NA + 4/7°C stress, respectively) for 3d. During the experiment, the environmental conditions in the climate chamber, except for temperature, were the same as normal growth conditions. The indicators were measured on the 3rd day after CA and cold stress treatment.

### Photosynthetic pigment concentrations and gas exchange measurements

The concentration determination of chlorophyll and carotenoids by using the method of Xu et al.,^29^ Healthy leaf samples were ground and extracted with 25 mL of acetone. Then, the mixture was centrifuged at room temperature at 4000 × g for 25 min.The absorbance was measured at 448 and 428 nm by using an ultraviolet spectrophotometer (UV-9100, LabTech, China). Chlorophyll and carotenoid concentrations were then calculated. The unit of Chl was expressed as mg g^−1^ (FM).

The gas exchange of citrus leaves was measured by using the Li-6400XT (Li-COR Inc., USA) from 9:00 to 11:00 based on the method of.^30^ Before the measurement, we conducted a 20 min light induction on the measured leaves. During the measurement process, the leaf chamber temperature was maintained at 25 ± 1°C, the relative humidity was 50 ± 5%, and the light intensity was 800 μmol m^−2^ s^−1^. Net photosynthetic rate (*P*_N_), stomatal conductance (*g*_s_), intercellular CO_2_ concentration (*C*_*i*_) and transpiration rate (T_r_) were automatically recorded by the instrument after equilibration to steady state.

### Chlorophyll a fluorescence elements measurements

Chlorophyll a fluorescence parameters were monitored by the plant efficiency analyzer (Handy PEA fluorimeter, Hansatech instruments Ltd. England). Prior to the measurement, the leaves were adapted to darkness at 25^◦^C for 20 min. The abbreviations, formulas, and definitions of the JIP experimental parameters used in this study are shown in Table S1.^31^

### Malondialdehyde and H_2_O_2_ contents measurements

The content of malondialdehyde (MDA) was measured using the thiobarbituric acid (TBA)-based colorimetric method, as described in He et al.,^32^ with slight modifications. The concentration of H_2_O_2_ in leaves was measured using the method of Xu et al.^33^ with almost no modification. The unit of MDA and H_2_O_2_ was expressed as μmol g^−1^ FM.

### Antioxidant enzyme activities measurements

Briefly, fresh green citrus leaves were ground in liquid nitrogen and extracted with 5 ml extraction buffer (50 mM K-phosphate buffer (pH 7.6) and 0.1 mMNa2-EDTA). The mixture was centrifuged at 4000×g for 25 min and then the antioxidant enzymes e activities were measured using the supernatant following the method of Mohammadrezakhani et al.^34^ The activity of SOD, APX and GR was expressed as units mg^−1^ protein, while the activity of CAT was expressed as nmol min^−1^ mg^−1^ protein.

### Rubisco and FBPase activity determinations

The activity of Rubisco and FBPase was measured using enzyme-linked immunosorbent assay (ELISA)^35,36^. Rubisco activity was measured using an ELX800 ELISA reader (BioTek, USA), while FBPase activity was measured using a ultraviolet spectrophotometer. The absorbance values of the samples at 450 nm and 340 nm wavelengths were measured, and their concentrations were calculated. The unit of Rubisco and FBPase was expressed as μmol g^−1^ s^−1^.

### Determination of abscisic acid and salicylic acid

HPLC (Thermo-separation product，model Spectra System P 2000, CA, USA) was used for abscisic acid and salicylic acid estimation. Detailed measurement steps according to the method by Agarwal et al.^37^

### Transcription levels of genes measurement

The gene transcription levels involved in the article were determined by Nanjing Huazhihai Biotechnology Co., Ltd. In briefly, a mass of approximately 0.2 g citrus leaves, which were previously and macerated in liquid nitrogen, was used for the extraction of total RNA using an RNAsimple Total RNA Kit (Tiangen Biotech, Co., Ltd., Beijing, China). The 2^−ΔΔCt^ method was used to calculate relative gene expression. The sequences of the primers used for quantitative real-time PCR were shown in Table S2

### Statistical analysis

The experiments were repeated three replicates containing three plants each. Data are analyzed by two-way analysis of variance (ANOVA) using the statistical software SPSS 17.0 (SPSS Inc. Ver.16, Chicago, IL, USA) and are presented as the means ± SEs. The differences were considered statistically significant at the 5% level by Duncan’s test. Figures were drawn with Graph Pad Prism version 7.05 for Windows (Graph Pad Software, San Diego, CA, USA).

## Results

### Photosynthetic pigments and gas exchange parameters

According to Table 1, CA slightly decreased chlorophyll content and increased carotenoid content in the citrus leaves compared to the NA group before exposure to cold stress. However, prior to the cold stress, there were no significant differences in the chlorophyll and carotenoid contents between the NA and CA groups. Moreover, the cold stress greatly reduced chlorophyll content and increased carotenoid content of CA and NA groups in citrus seedlings. The chlorophyll content in the CA + cold stress group was 23.59% higher and the carotenoid content was 5.81% lower than that of the NA + cold stress group, respectively. In addition, the chlorophyll/carotenoid ratio was progressively decreased by 4/7°C cold stress in both the CA and NA plants.

**Table 1.** Effects of cold acclimation on chlorophyll and carotenoid concentrations in citrus seedling leaves under cold stress. Note. The data in the table is the average value of three replicated samples. Different letters indicate significant differences among different treatments at *P* < .05.

Treatments	Chlorophyll		Carotenoid		Chlorophyll/carotenoid
	Mean (mg g^−1^ FM)	Percentage of NA (%)	Mean (mg g^−1^ FM)	Percentage of NA (%)	
NA	3.21 ± 0.12a	/	0.82 ± 0.02c	/	3.91 ± 0.13a
CA	3.17 ± 0.14a	98.75	0.81 ± 0.01c	98.78	3.91 ± 0.11a
NA + 4/7°C	2.17 ± 0.09c	67.60	0.91 ± 0.03a	110.97	2.38 ± 0.09c
CA + 4/7°C	2.84 ± 0.11b	88.47	0.86 ± 0.02b	104.87	3.30 ± 0.12b

**Figure 1.**
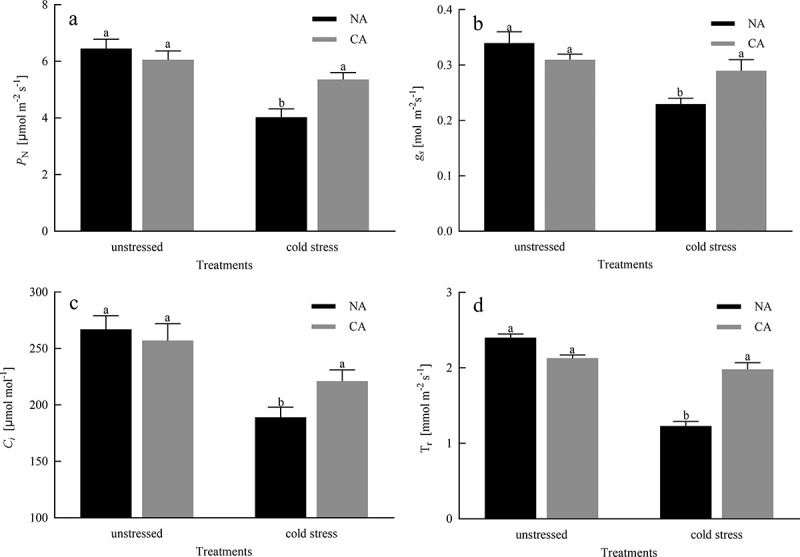
Effects of cold acclimation on *P*_N_, *C*_*i*_, *g*_s_ and T_r_ under cold stress in citrus seedling leaves. Note. *P*_N_ – net photosynthetic rate, *g*_s_ – stomatal conductance, *C*_*i*_ – intercellular CO_2_ concentration, T_r_ – transpiration rate, NA – without cold acclimation, CA – cold acclimation. The data in the figure is the average value of three replicated samples. Different letters indicate significant differences among different treatments at *P* < .05.

The gas exchange parameters including *P*_N_, *C*_*i*_, *g*_s_, and T_r_ were slightly reduced in CA treatment compared to that in the NA group before exposure to cold stress (Figure 1a–d). However, the reductions were not more pronounced in NA or CA seedlings. Under cold stress, CA treatment decreased the negative effects of extreme temperatures on these parameters and showed higher levels of *P*_N_, *C*_*i*_, *g*_s_, and T_r_ by 61%, 73%, 9% and 72%, respectively, compared to that in the NA group. There were significant differences between the CA + cold stress group and the NA + cold stress group regarding these parameters of gas exchange.

### Rubisco and FBPase activities and transcription levels of *rbcL* and *rbcS* genes

**Figure 2.**
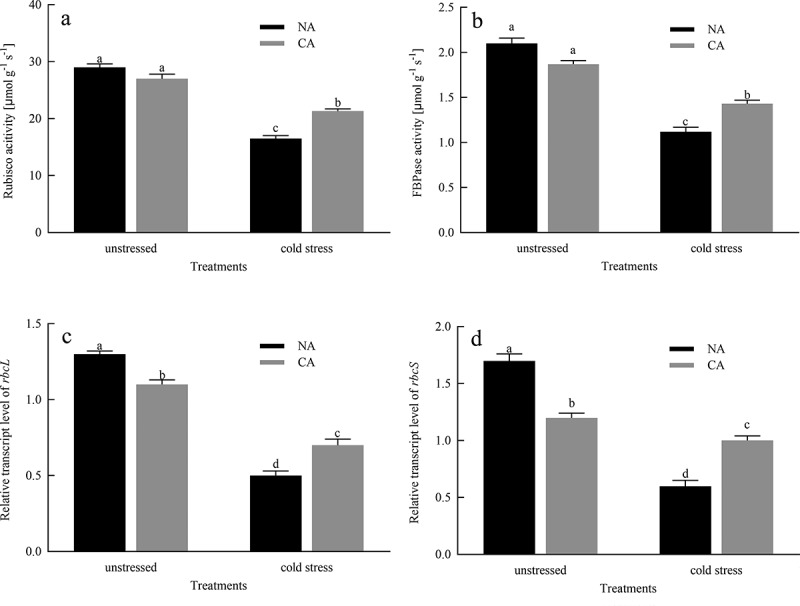
Effects of cold acclimation on the activities of Rubisco and FBPase and the relative transcription levels of *rbcL* and *rbcS* genes under cold stress in citrus seedlings leaves. Note. Rubisco – ribulose-1,5-bisphosphate carboxylase/oxygenase, FBPase – Fructose1,6-bisphosphatase, *rbcS* – photosynthesis related gene, *rbcL* – photosynthesis related gene, NA – without cold acclimation, CA – cold acclimation. The data in the figure is the average value of three replicated samples. Different letters indicate significant differences among different treatments at *P* < .05.

Compared to the NA group before cold stress, CA treatment slightly reduced the activities of Rubisco and FBPase in citrus leaves, but did not exhibit significant cold damage (Figure 2a, b). Moreover, cold stress reduced the Rubisco and FBPase activities of CA and NA plants. The difference in Rubisco and FBPase activity between the CA group and the NA group under cold stress widened greatly. The transcription levels of *rbcL* and *rbcS* in citrus leaves treated with CA immensely decreased by 15.38% and 29.41%, respectively, compared to the NA group before cold stress (Figure 2c, d). In addition, the cold stress significantly decreased the transcript levels of *rbcL* and *rbcS* in both NA and CA plants. However, compared to the NA + cold stress group, the transcript levels of *rbcL* and *rbcS* were higher in the CA + cold stress group by 28.57% and 40.09%, respectively.

### Chlorophyll a fluorescence parameters

**Figure 3.**
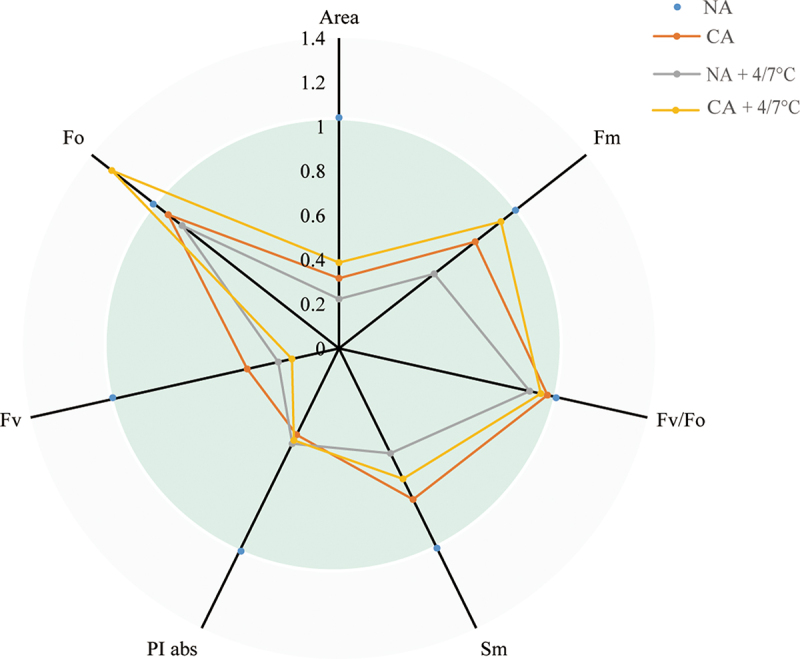
Effects of cold acclimation on chlorophyll *a* fluorescence parameters under cold stress in citrus seedlings leaves. Note. Area – total complementary area between the fluorescence, F_o_ – minimal fluorescence, F_v_ – maximal variable fluorescence, PI_abs_ – performance index (potential) for energy conservation from exciton to the reduction of intersystem electron acceptors, S_m_ – normalized total area above the OJIP curve, F_v_/F_o_ – ratio of rate constants for photochemical and nonphotochemical use of RC excitation energy, F_m_ – maximal fluorescence, when all PSII RCs are closed, NA – without cold acclimation, CA – cold acclimation. The data in the figure is the average value of three replicated samples.

As shown in Figure 3，The Area, F_o_, F_v_, PI_abs_, S_m_, F_v_/F_o_ and F_m_ values in the CA and NA groups without cold stress were significantly different, with the values of CA group significantly higher than those in the NA group. Old stress reduced chlorophyll *a* fluorescence parameters in both CA and NA plants. Although F_v_ and PI_abs_ values in the CA + cold stress group were smaller than those in the NA + cold stress group, there was no significant difference. Moreover, the value of Area, F_o_, S_m_, F_v_/F_o_ and F_m_ in the CA + cold stress group was significantly higher than those in the NA + cold stress group.

**Figure 4.**
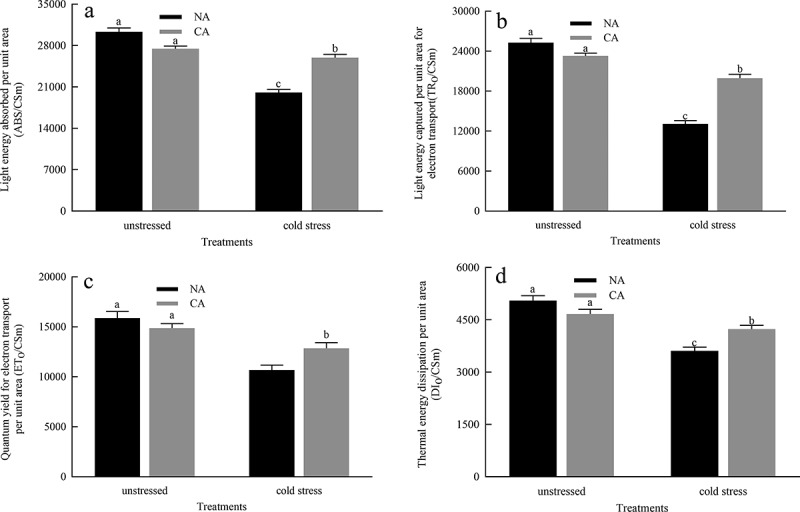
Effects of cold acclimation on phenomological energy flux per unit area under cold stress. Note. ABS/CS_m_ – light energy absorbed per unit area, TR_o_/CS_m_ – light energy captured per unit area for electron transport, ET_o_/CS_m_ – quantum yield for electron transport per unit area, DI_o_/CS_m_ – thermal energy dissipation per unit area, NA – without cold acclimation, CA – cold acclimation. The data in the figure is the average value of three replicated samples.

Phenomenological energy fluxes i.e. ABS/CSm, TRo/CSm, ETo/CSm and DIo/CSm are shown in Figure 4. In phenomenological fluxes per cross-section and the leaf model, The ABS/CSm, TR/CSm, ETo/CSm, and DIo/CSm of plants in the CA group were significantly affected compared to those in the NA group. The cold stress treatment significantly decreased ABS/CSm, TR/CSm, ETo/CSm, and DIo/CSm. However, ABS/CSm, TR/CSm, ETo/CSm, and DIo/CSm were greatly higher in the CA+ cold stress group, compared to those in the NA+ cold stress group.

### H_2_O_2_ and MDA contents

**Figure 5.**
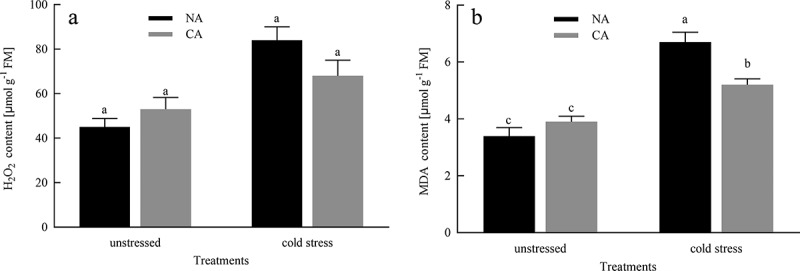
Effects of cold acclimation on H_2_O_2_ and MDA under cold stress in citrus seedling leaves. Note. H_2_O_2_ – hydrogen peroxide, MDA – malondialdehyde, NA – without cold acclimation, CA – cold acclimation. The data in the figure is the average value of three replicated samples. Different letters indicate significant differences among different treatments at *P* < .05.

CA slightly increased the production rate of H_2_O_2_ content in citrus leaves (Figure 5). However, before cold stress treatment, there was no significant difference in the production rate of H_2_O_2_ content between the NA group and the CA group. In addition, compared to the NA group before cold stress, the MDA content in the CA group slightly increased. The contents of H_2_O_2_ and MDA in the CA and NA plants were greatly increased under cold stress. However, the contents of H_2_O_2_ and MDA in the CA + cold stress group decreased by 19.04% and 22.38%, respectively, in comparison to the NA + cold stress group.

### Antioxidant enzyme activity

**Figure 6.**
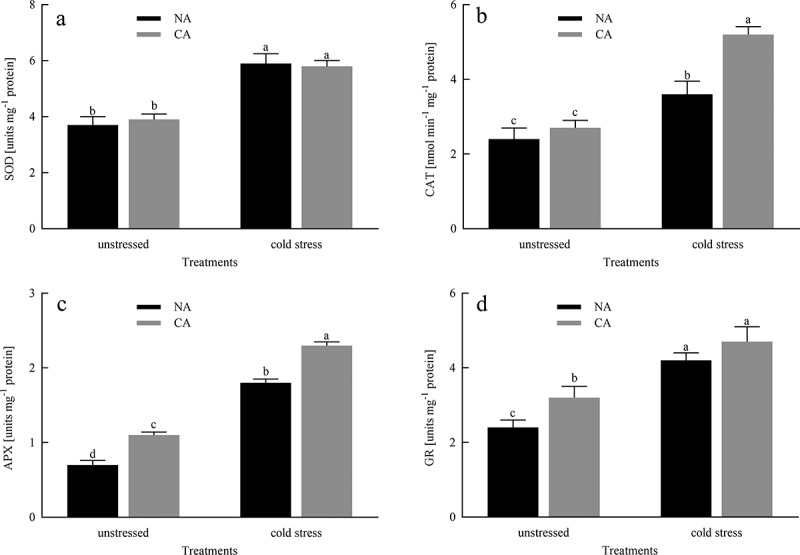
Effects of cold acclimation on SOD, CAT, APX and GR activity under cold stress in citrus seedling leaves. Note. SOD – superoxide dismutase, CAT – catalase, APX – ascorbate peroxidase, GR – glutathione reductase, NA – without cold acclimation, CA – cold acclimation. The data in the figure is the average value of three replicated samples. Different letters indicate significant differences among different treatments at *P* < .05.

Compared to the NA group before cold stress, CA slightly increased the activities of SOD and CAT in citrus leaves, but there was no significant difference in the activities of SOD and CAT between the NA group and the CA group (Figure 6). Moreover, CA significantly increased the activities of APX and GR, compared to the NA group before exposure to cold stress. The activities of SOD, CAT, APX, and GR in the CA and NA plants were significantly increased under cold stress. However, compared with NA + cold stress group, the CA + cold stress group showed significantly higher CAT, APX, and GR activities, while SOD activities showed relatively stable.

### Transcription levels of genes encoding antioxidant enzymes

**Figure 7.**
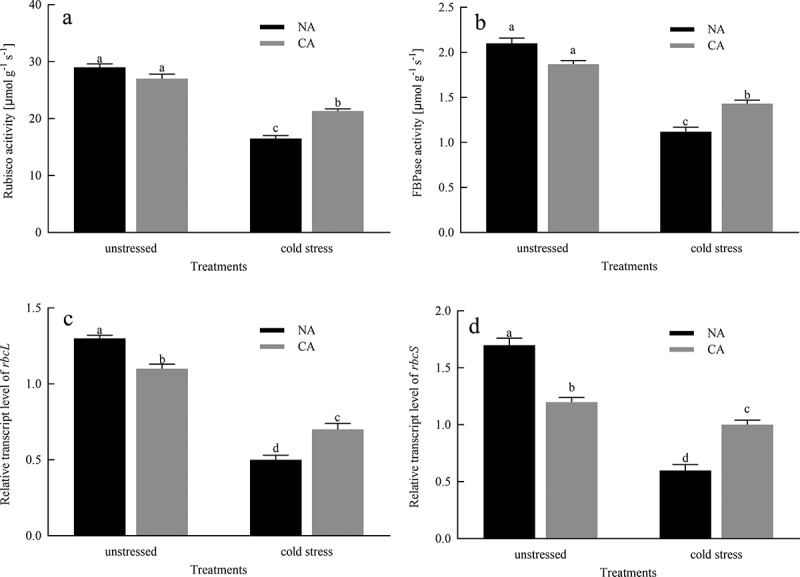
Effects of cold acclimation on the relative transcription levels of *CuZnSOD1*, *CAT1*, *APX*, *GR* genes in citrus seedlings leaves under cold stress. Note. *CuZnsod1* – encoding gene of SOD, *CAT1* – encoding gene of CAT, *APX* – encoding gene of *APX*, *GR* – encoding gene of GR, NA – without cold acclimation, CA – cold acclimation. The data in the figure is the average value of three replicated samples. Different letters indicate significant differences among different treatments at *P* < .05.

As shown in Figure 7, only 5.26% of the *CuZnSOD1* transcript level was reduced in the CA group, compared to that in the NA group before cold stress. Cold stress greatly increased the transcription level of *CuZnSOD1*, and compared to the NA + cold stress group, the transcription level of *CuZnSOD1* in the CA+ cold stress group was pronouncedly higher. Moreover, CA significantly increased the *CAT1, APX* and *GR* transcript levels compared to the NA group before cold stress. The cold stress greatly increased *CAT1*, *APX* and *GR* transcript levels in both CA and NA plants, with the transcription levels of *CAT1*, *APX* and *GR* in CA + cold stress group being 21.74%, 31.92%, and 24.07% higher than those in NA + cold stress group, respectively.

### ABA and SA contents

**Figure 8.**
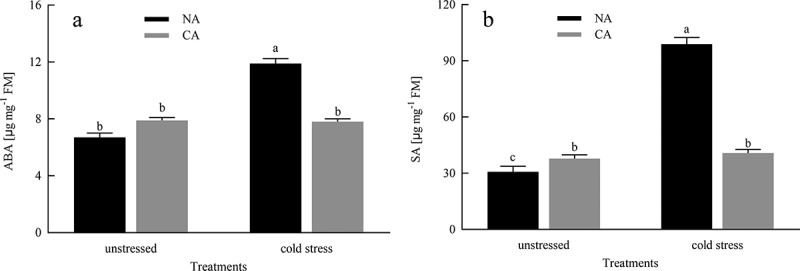
Effects of cold acclimation on ABA and SA in citrus seedlings leaves under cold stress. Note. ABA – abscisic acid, SA – salicylic acid, NA – without cold acclimation, CA – cold acclimation. The data in the figure is the average value of three replicated samples. Different letters indicate significant differences among different treatments at *P* < .05.

As shown in Figure 8, prior to cold stress treatment, the citrus leaves in the CA group exhibited a slight increase in ABA content compared to the NA group, although the difference was not statistically significant. Conversely, when subjected to cold stress conditions, the ABA content in the NA group significantly increased, and the CA + cold stress group demonstrated an 52.56% increase in ABA content compared to the NA + cold stress group. Regarding SA, prior to cold stress treatment, the SA concentration in the CA group exhibited a statistically significant elevation compared to the NA group. Following exposure to cold stress, the SA concentration in NA plants experienced a significant augmentation, and the ABA concentration in the NA + cold stress group was 1.42 times higher than that in the CA + cold stress group.

## Discussion

Chlorophylls (Chl) are essential for light-harvesting during photosynthesis.^38^ A drop in temperature, without freezing, causes a serious slowdown or even degradation of chlorophyll synthesis, which is not conducive to the normal growth and development of plants. In this experiment, we confirmed that CA pretreatment reduced the degradation of chlorophyll and carotenoid content induced by cold stress (Table 1), indicating that CA can effectively stimulate chlorophyll synthesis and alleviate the harsh effects of stress factors under cold stress conditions. Photosynthesis is the most fundamental physiological process in plants, and it is an important source of power for its growth, development, and physiological metabolic activities. However, any biotic and abiotic stress that changes the components of photosynthetic will lead to the decline of *P_N_*.^39^ Our study showed that citrus seedlings subjected to cold stress exhibit a significant decrease in photosynthesis, but plants with CA pretreated exhibit an increase in photosynthesis (Figure 1). The decrease in *P*_N_ under cold stress may be due to the disruption of the dynamic balance between *g*_s_ and *C*_*i*_. When *g*_s_ and *C*_*i*_. decrease together, the stomatal conductivity is a key factor limiting *P_N_*.^40^ On the other hand, under cold stress, *P*_N_ increased in citrus seedlings treated with CA, which may be the result of simultaneous positive induction of *g*_s_ and *C*_*i*_. Photosynthesis disruption by cold stress is also reflected in photosynthetic enzymes. Rubisco is a rate-limiting enzyme for the carboxylation of RuBP, and is also one of the main centers under any stress condition.^10^ In current study, cold stress significantly reduced the activity of RuBP leading to the downregulation of photosynthesis. However, the CA treatment before imposing cold stress greatly elevated the activity of RuBP (Figure 2), which improves photosynthetic CO_2_ assimilation rates. In plants, FBPase activity is the main stromal diphosphatase that monitors photosynthesis at low temperatures, and its activity is closely related to the redox state of the chloroplast^41^. According to Daems et al^42^ confirmed that FBPase activity was inhibited in chilling-stressed plants. Our study showed that in cold-stressed citrus plants, CA increased FBPase activity, suggesting that CA alleviates inhibitory effects of FBPase activity in citrus seedlings under cold conditions. Our study also measured the transcript levels of two genes that encode Rubisco, namely *rbcL and rbcS*, which were reduced in citrus leaves under cold stress, but the decrease was mitigated by CA treatment before cold stress (Figure 2). Therefore, CA treatment before cold stress enhanced the transcript levels of *rbcL* and *rbcS*, thereby enhancing the ability of citrus seedlings to resist cold stress and reducing the inhibition to photosynthetic capacity.

Chlorophyll fluorescence has emerged as a vital tool for assessing the photosynthetic process/systems in plants under various stressful conditions.^16^ An extreme-stress condition result in significant photoinhibition of the PSII reaction center.^43^ F_v_/F_m_ is the most widely used photo-oxidative stress markers. In healthy leaves, the value of Fv/Fm is very stable, ranging from 0.80 to 0.83, which is also related to the maximum quantum yield of photosynthesis.^44^ When the value of Fv/Fm is lower than 0.75, it indicates that PSII is damaged, photoinhibition, and therefore it is very useful stress marker.^33^ In our experiment, cold stress greatly reduced the value of F_v/_F_m_, but CA treatment before the cold stress protects the PSII form photoinhibition (Figure 3). Area parameter represents the total complementary area between the fluorescence induction curve and F_m_^45^ which was higher in CA+ cold plants than in only cold treated plants (Figure 3). The Area is relative to the pool size of the electron acceptors QA on the reducing side of PSII. According to previous reports, the decrease in area parameters will be due to the blocking of electron transfer from RC to the quinone pool^46^. Hence, CA treatment helps to increase the electron transfer from the RC to the quinone pool before cold stress. PI_abs_ is calculated on energy absorption basis^47^ and PI_abs_ value is higher in the plant under CA+cold group (Figure 3). PI_abs_ is increased due to increased activity of the RC so the overall activity of the RC is increased. Sm, assessing of the electron transporter PQ pool between PS II and PSI,^48^ is also increase in the plant under CA+ cold stress group displayed an increased electron transport between these photosystems. As a result of photosynthetic electron transport, precise rhythms of photosynthesis are maintained and an optimal energy flow is ensured, which contributes to plant growth and development as well as stress responses.^49^

The decreases in ABS/CSm, DIo/CSm, TRo/CSm, and ETo/CSm suggested substantial interference with the active reaction centers of PSII under cold without CA treatment. However, the plants under CA+ cold group have the higher values of ABS/CSm, DIo/CSm, TRo/CSm, and ETo/CSm, which may be due to the help of CA in maintaining the balance of energy transfer from the light harvesting complex to the chlorophyll reaction center in the photosystem.

H_2_O_2_ is an oxidant and is involved in the production of many other ROS.^50^ The amount of ROS gives us information about the status of the imbalance during a stress response. MDA is the final product of membrane lipid peroxidation, which is used to examine the oxidative damage in the cell membranes.^51^ Our research results indicated that cold stress caused damage to the membrane of citrus leaves and increased the content of H_2_O_2_ (Figure 5). The plants in CA + cold stress group lowered this trend by a decrease of MDA and H_2_O_2_ levels, compared to that in the NA + cold stress group, suggesting that CA treatment has a certain protective effect on membrane damage under cold stress induced conditions. This conclusion was similar to the research results of Ruelland et al.^52^ To prevent from oxidative stress and maintain cell activity, plants respond to stress by regulating the expression of scavenger enzymes (SOD, CAT, APX, GR) in the cell membrane system to prevent oxidative damage.^53^ SOD play the most potential role in protecting cell membrane degradation and scavenging ROS, which is the first defense mechanism of the antioxidant system.^54^ By converting O_2_^−^ to H_2_O_2_, which is then rapidly detoxified by CAT by transferring H_2_O_2_ to H_2_O and O_2_^55^, and thereafter, CAT, POD and APX. The above enzymes effectively enhance the scavenging of ROS and free radicals, thereby protecting seedlings from cell damage caused by oxidative stress. Our research showed that under cold stress conditions, the activities of CAT, SOD, APX, and GR significantly increased, while the pre-treatment of CA actively stimulated the activity of these enzymes to provoke the cold resistance of citrus seedlings (Figure 6), indicating that the cumulative effect of all antioxidant enzymes promotes the efficacy of scavenger by increasing antioxidant functions, thereby playing a protective role in the cell membrane. Our study also investigated the transcript levels of redox enzymes-related genes such as *CuZnSOD1, CAT1, APX*, and *GR*, which were higher in the CA+ cold stress group, compared to that in the NA+ cold stress group, suggesting that CA treatment prior to cold stress enhances redox enzymes-related genes expression levels and prevents antioxidant enzymes inactivation, thus improving cold tolerance of citrus seedlings.

## Conclusion

**Figure 9.**
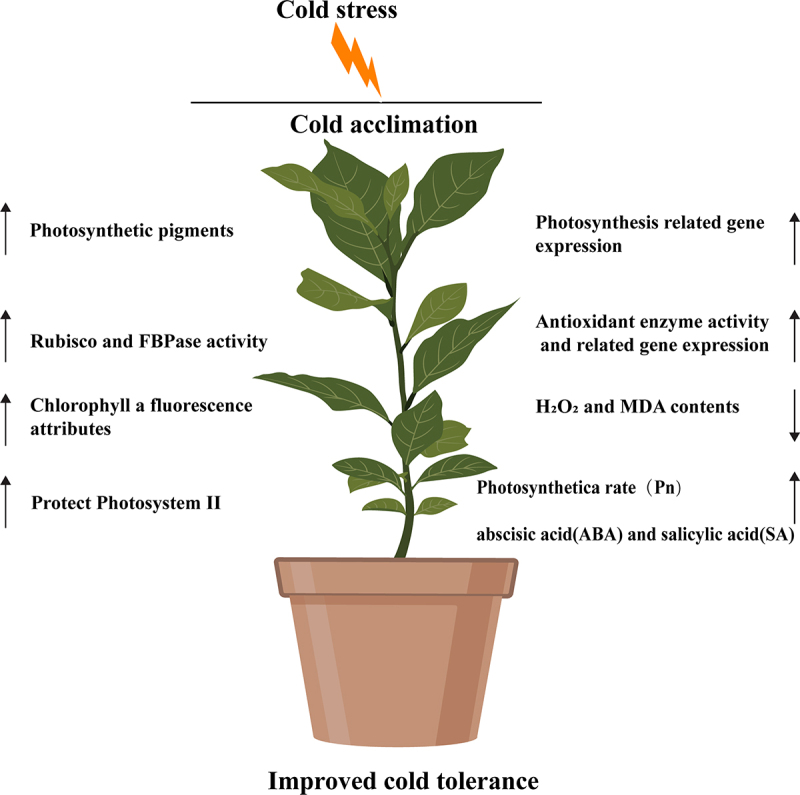
Schematic representation of the positive role of cold acclimation (CA) on cold tolerance in citrus plant.

The model of cold acclimation treatment before cold stress improving the cold resistance of citrus seedlings was shown in Figure 9. The conclusions of this study indicated that exposure to cold stress resulted in an increase in the concentration of photosynthetic pigments and enhanced the activities of Rubisco and FBPase, as well as led to an upregulation of transcription levels of photosynthetic related genes (*rbcL* and *rbcS*). The treatment of cold acclimation significantly improved the photochemical efficiency of photosystem II (PSII), particularly the maximum quantum efficiency (F_v_/F_m_) of PSII, and enhanced the efficiency of electron transfer. Moreover, cold acclimation alleviated oxidative stress damage to the cell membrane by inhibiting the concentrations of H_2_O_2_ and MDA, enhancing the activities of superoxide dismutase (SOD), catalase (CAT), ascorbate peroxidase (APX), and glutathione reductase (GR), and increasing the expression levels of antioxidant enzyme genes (*CuZnSOD1, CAT1, APX*, and *GR*). Additionally, cold acclimation also increased the contents of abscisic acid (ABA) and salicylic acid (SA) in plants under cold stress.

In summary, when subjected to cold stress at temperatures below 4/7°C, the process of cold acclimation demonstrates its potential in mitigating the photoinhibition of PSII and oxidative harm to citrus leaves. This is achieved through the enhancement of photosynthetic apparatus performance, augmentation of antioxidant enzyme and photosynthetic enzyme activities, optimization of energy distribution within PSII reaction centers, and reduction in the accumulation of reactive oxygen species (ROS) and malondialdehyde (MDA).
